# Conditional Knock out of High-Mobility Group Box 1 (HMGB1) in Rods Reduces Autophagy Activation after Retinal Detachment

**DOI:** 10.3390/cells10082010

**Published:** 2021-08-06

**Authors:** Bing X. Ross, Lin Jia, Dejuan Kong, Tiantian Wang, Heather M. Hager, Steven F. Abcouwer, David N. Zacks

**Affiliations:** 1 Kellogg Eye Center, Department of Ophthalmology, University of Michigan, 1000 Wall St, Ann Arbor, MI 48105, USA; bxu@med.wayne.edu (B.X.R.); ljia@med.umich.edu (L.J.); dejuank@med.umich.edu (D.K.); tiantiaw@med.umich.edu (T.W.); hlindner@med.umich.edu (H.M.H.); sabcouwe@med.umich.edu (S.F.A.); 2 Department of Ophthalmology, Xiangya Hospital, Central South University, Changsha 410008, China

**Keywords:** retinal detachment, HMGB1, photoreceptor, autophagy, apoptosis

## Abstract

After retinal detachment (RD), the induction of autophagy protects photoreceptors (PR) from apoptotic cell death. The cytoplasmic high-mobility group box 1 (HMGB1) promotes autophagy. We previously demonstrated that the deletion of HMGB1 from rod PRs results in a more rapid death of these cells after RD. In this work, we tested the hypothesis that the lack of HMGB1 accelerates PR death after RD due to the reduced activation of protective autophagy in the retina after RD. The injection of 1% hyaluronic acid into the subretinal space was used to create acute RD in mice with a rhodopsin-Cre-mediated conditional knockout (cKO) of HMGB1 in rods (HMGB1Δrod) and littermate controls. RD sharply increased the number of apoptotic cells in the outer nuclear layer (ONL), and this number was further increased in HMGB1Δrod mouse retinas. The activation of autophagy after RD was reduced in the HMGB1Δrod mouse retinas compared to controls, as evidenced by diminished levels of autophagy regulatory proteins LC3-II, Beclin1, ATG5/12, and phospho-ATG16L1. The cKO of HMGB1 in rods increased the expression of Fas and the Bax/Bcl-2 ratio in detached retinas, promoting apoptotic cell death. In conclusion, endogenous HMGB1 facilitates autophagy activation in PR cells following RD to promote PR cell survival and reduce programmed apoptotic cell death.

## 1. Introduction

Autophagy is a highly conserved physiological process that sequesters and delivers intracellular material such as organelles and long-lived proteins to lysosomes for degradation and recycling [1,2,3,4]. Under stress conditions such as nutrient deprivation, hypoxia, or exposure to toxic materials, autophagy can be induced to conserve cellular energy and promote cell survival [1]. As a cellular stress response pathway, autophagy also contributes to the innate immune defense against invading pathogens, likely related to the starvation signal resulting from competition for intracellular nutrients [5]. Autophagy has subsequently evolved to actively participate in multiple aspects of innate and adaptive immunity and modulation of inflammation [5,6].

In the retina, autophagy is important in maintaining normal homeostasis as well as in reducing cell death during periods of stress [7,8,9]. We have previously shown that retinal detachment (RD) results in the activation of autophagy in photoreceptor (PR) cells [10]. Blocking autophagy pharmacologically results in greater PR cell death after RD, primarily through apoptosis [10]. In prolonged detachment, there is a reduction in the activation of autophagy, and a shift from cell survival to cell death [11]. These results suggest that in the early stages of RD, the activation of autophagy serves as a critical mechanism for enhancing PR survival. The molecular mechanisms responsible for this activation of autophagy after RD are still poorly understood.

HMGB1 is a multifunctional protein with compartment-specific functions. Under baseline conditions, HMGB1 resides in the nucleus, acting as a nonhistone DNA-binding protein to facilitate DNA transcription and repair [12]. During periods of stress, HMGB1 can be mobilized to the cytoplasm to regulate autophagy flux [13,14,15]. When secreted or released into the extracellular space, HMGB1 can function as a damage-associated molecular pattern (DAMP) molecule to activate innate immunity and inflammation [16]. Our previous studies have demonstrated that the conditional knockout (cKO) of HMGB1 in rods accelerates PR cell death following RD [17]. We have demonstrated that, at least at the early time points post-detachment (3 d or 7 d post-RD), deletion of HMGB1 in rods does not affect microglia/macrophages mobilization into the stressed PR layer or the subretinal space, nor does it affect HMGB1 levels in the extracellular space, suggesting that the extracellular functions of HMGB1 as a DAMP signal molecule (non-cell-autonomous function) may not be the dominant factor that drives accelerated PR cell death after RD [17].

Upon starvation, HMGB1 translocates from the nucleus to the cytoplasm [14,17], where it helps control the switch between autophagy and apoptosis [15]. Loss of HMGB1 can reduce autophagy activation under oxidative stress conditions both in vivo and in vitro [13]. We therefore explored the cell-autonomous functions of HMGB1 in regulating PR cell death after RD, and in particular, whether HMGB1 played a role in the activation of autophagy on the detached retina. Our results demonstrate that the depletion of HMGB1 in rod PRs results in greater loss of these cells during RD and suggest that intracellular HMGB1 is important in the upstream activation of autophagy in rod PRs following RD. This study thus demonstrates the important role of HMGB1 in the response of PRs to retinal detachment and confirms the cell-autonomous functions of HMGB1 in promoting PR cell survival.

## 2. Materials and Methods

### 2.1. Animals

All mouse experiments and procedures were performed in accordance with The Association for Research in Vision and Ophthalmology (ARVO) Statement for the Use of Animals in Ophthalmic and Vision Research and approved by the Animal Care and Use Committee of the University of Michigan. Mice with conditional knockout of HMGB1 in rod PR cells (denoted as HMGB1Δrod) were generated, as previously reported [17], and used at 8–12 weeks of age, with littermates as control. All mice were housed in the vivarium at University of Michigan Kellogg Eye Center under a 12 h light/dark cycle.

### 2.2. Mouse Model of Experimental Retinal Detachment

Retinal detachment was produced, as previously stated [17]. Briefly, ketamine (50 mg/kg) and xylazine (5 mg/kg) diluted in 1× phosphate buffered saline (PBS) were used to induce anesthesia in mice, and topical tropicamide (1%) and phenylephrine (2.5%) to dilute their pupils. A sclerotomy was created at approximately 1 mm posterior to the limbus with a 30-gauge needle. A microsyringe (NANOFIL-100 injector system; World Precision Instruments, Sarasota, FL, USA) loaded with 1% hyaluronic acid (Healon; Abott Medical Optics, Santa Ana, CA, USA) was attached to a 35-gauge needle. The tip of the needle was inserted through the sclerotomy, passed through the vitreous cavity, and advanced into the subretinal space. Hyaluronic acid was subsequently injected into the subretinal space to detach approximately one-half of the neurosensory retina from the underlying retinal pigment epithelium. Naïve retinas without any manipulation of either eye was used as attached retina controls.

### 2.3. Blockage of Autophagy Flux

Leupeptin was used to block autophagic flux. Leupeptin hemisulfate (cat. L2884; Sigma-aldrich, Saint Louis, MO, USA) was dissolved in sterile PBS and intraperitoneally (i.p.) injected into mice at a dose of 40 mg/kg at 4 h before sample collection to allow the detection of autophagic flux. Sterile PBS was used as vehicle control.

### 2.4. Western Blotting

Mice were euthanized at desired time points after RD. Eyes were enucleated and retinas were dissected out and lysed in a RIPA buffer (cat. 89901; Thermo Fisher Scientific, Waltham, MA, USA) containing a protease and phosphatase inhibitor cocktail (cat. 78441; Thermo Fisher Scientific, Waltham, MA, USA). The whole retina lysates were centrifuged at 2000× *g* for 10 min at 4 °C and supernatant was collected for protein concentration measurement using DC Protein Assay (cat. 5000116; Bio-Rad, Hercules, CA, USA). Equal amounts of proteins were loaded onto 4–15% SDS-PAGE (cat. 4561086; Bio-Rad, Hercules, CA, USA) for electrophoresis. The proteins were then transferred to polyvinylidene fluoride membranes (cat.1620177; Bio-Rad, Hercules, CA, USA), which were then blocked and incubated with primary antibodies overnight at 4 °C under gentle shaking. After three washes, the membranes were incubated with peroxidase-conjugated secondary antibody (cat. G-21234; Thermo Fisher Scientific, Waltham, MA, USA) for 2 h at room temperature (RT). Signals were revealed by using enhanced chemiluminescence (SuperSignal West Dura Substrate, cat. 34075; Thermo Fisher Scientific, Waltham, MA, USA) and detected by cSeries Capture Software (c500; Azure Biosystems, Dublin, CA, USA). Density quantification was performed with ImageJ software (National Institutes of Health, Bethesda, MD, USA).

Primary antibody list: anti-HMGB1 (cat. PA1-10926; Thermo Fisher Scientific, Waltham, MA, USA), anti-phospho-ATG16L1 (Ser278, cat. Ab195242; Abcam, Cambridge, MA, USA), anti-ATG5 (cat. NB110-53818; Novus Biologicals, Littleton, CO, USA). The following antibodies were purchased from Cell Signaling Technology (Danvers, MA, USA): anti-LC3-I/II (cat. 4108), anti-Beclin1 (cat. 3495), anti-phospho-ULK1 (Ser757, cat. 14202), anti-phospho-ULK1 (Ser555, cat. 5869), anti-phospho-ATG14 (Ser29, cat. 92340), anti-phospho-Erk1/2 (Thr202/Tyr204, cat. 9101), anti-Erk1/2 (cat. 9102), anti-Bcl-2 (cat. 3498), and anti-Bax (cat. 2772). Anti-glyceraldehyde 3-phosphate dehydrogenase (GAPDH, cat. AM4300; Applied Biosystems, Foster City, CA, USA) was used as a loading control.

### 2.5. Quantitative Real-Time PCR (qRT-PCR)

Total RNA was extracted using RNeasy Mini kit (cat. 74104; Qiagen, Hilden, Germany), according to the manufacturer’s instructions. After measuring the concentration of RNA with a NanoDrop One Spectrophotometer (cat. 701058108; Thermo Fisher Scientific, Waltham, MA, USA), 500 ng total RNA was used to reverse-transcribe to cDNA by using the SuperScript III Reverse Transcriptase Kit (cat. 18080093; Thermo Fisher Scientific, Waltham, MA, USA), according to the manufacturer’s instructions. Quantitative RT-PCR was performed with SYBR Green PCR Master Mix (cat. 4309155; Applied Biosystems, Foster City, CA, USA), according to the manufacturer’s instructions. The mRNA expression level of each target gene was normalized to the expression level of RPL19. The results of the PCR assay represent the means of four independent RNA preparations in each group.

The mouse primer pair sequences used are as follows: *Fas*, 5′-GGAGGCGGGTTCGTGAAACTGA-3′ and 5′-TTCCCTTCTGTGCATGGGGCG-3′; *TLR2*, 5′-TGCTTCGTTGTTCCCTGTGT-3′ and 5′-AGAGCTGGCGTCTCCATAGT-3′; *CCL2*, 5′-CGTTAACTGCATCTGGCTGA-3′ and 5′-AGCACCAGCCAACTCTCACT-3′; *pro-IL-1β*, 5′-GCCCATCCTCTGTGACTCAT-3′ and 5′-AGGCCACAGGTATTTTGTCG-3′; *CXCL10*, 5′-TGAGCTAGGGAGGACAAGGA-3′ and 5′-GGATGGCTGTCCTAGCTCTG-3′; *CCL3*, 5′-AGAGTCCCTCGATGTGGCTA-3′ and 5′-GTGTAGAGCAGGGGCTTGAG-3′; *RPL19*, 5′-ATGCCAACTCCCGTCAGCAG-3′ and 5′-TCATCCTTCTCATCCAGGTCACC-3′.

### 2.6. Cell Death Evaluation

Mouse eyes were enucleated and fixed with 4% paraformaldehyde in PBS for 2 h. After washing with PBS twice for 10 min, corneas and lens were dissected out and the remaining posterior eyecups were sequentially passed through an increasing gradient concentration of sucrose in PBS for cryo-protection: 5% sucrose at RT for 1 h, 10% sucrose at RT for 1 h, and 20% sucrose at 4 °C overnight. A mixture of 20% sucrose and an optimal cutting temperature (OCT) compound (Tissue Tek 4583, Sakura Finetek, Tokyo, Japan) at a 1:1 ratio was used to embed the eyecups. Ten-micron sections that cross the optic nerve head were obtained and used to perform the terminal deoxynucleotidyl transferase-mediated dUTP nick-end labeling (TUNEL) assay (DeadEnd™ Fluorometric TUNEL System. cat. G3250; Promega, Madison, WI, USA), according to the manufacturer’s instructions. Nuclei were counterstained with 4′,6-diamidino-2-phenylindole (DAPI). Images were captured with a Leica DM6000 microscope (Leica Microsystems, Wetzlar, Germany) with fixed detection gains.

Imaris software (Oxford Instruments plc., UK) was used for the quantification of the TUNEL positive cells in the ONL in the attached and detached retinas, from the optic nerve head to the tip of the retinal periphery in a masked fashion. Two individuals who were masked to the experimental groups performed the cell count independently. The number of TUNEL positive cells from 3 non-continuous sections that crossed the optic nerve head were averaged to obtain the final number for each eye. Each group contains 5 independent eyes.

### 2.7. Statistical Analyses

Data were presented as mean ± SD and analyzed with Prism 9 software (GraphPad Software, Inc., San Diego, CA, USA). Unpaired Student’s *t*-test was used for comparison between two groups, and one-way ANOVA for a comparison of more than two groups, followed by Bonferroni post hoc correction. Significance was accepted at *p* < 0.05.

## 3. Results

### 3.1. Conditional Knockout of HMGB1 in Rods Leads to Increased PR Cell Death

**Figure 1:**
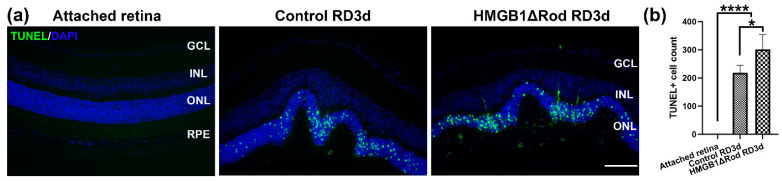
Loss of HMGB1 in rods increases apoptosis of photoreceptor cells after retinal detachment (RD). (**a**) TUNEL Rod mice at RD 3 d. Nuclei were counterstained with 4′,6-diamidino-2-phenylindole (DAPI). Green: TUNEL positive cells, Blue: DAPI. Scale bar: 100 µm. GCL: ganglion cell layer, INL: inner nuclear layer, ONL: outer nuclear layer, RPE: retinal pigment epithelium. (**b**) Graph shows the quantification of TUNEL positive cells in the ONL in the attached and detached retinas from (**a**). Three non-continuous sections that cross the optic nerve head were used for the quantification of each eye. Each group contains five independent eyes. Data are plotted with mean ± s.d. * *p* < 0.05, **** *p* < 0.0001 (ANOVA).

Our previous study showed that the lack of HMGB1 in rods resulted in the significant thinning of the ONL at one month post-RD [17]. The majority of PR cells die through apoptosis, and apoptotic cell numbers peak at three days post-RD (dprd) in a rodent RD model [18,19,20,21,22]. Therefore, we chose three dprd to evaluate PR cell death in HMGB1Δrod versus littermate controls. As shown in Figure 1a,b, RD caused increased TUNEL positive cells in the ONL, and the number of TUNEL positive cells were significantly higher in the HMGB1Δrod RD group compared to the control RD group (38.4% increase).

These data demonstrate that cKO of HMGB1 in rods causes more PR cell death at an early time point following RD.

### 3.2. Lack of HMGB1 Impairs RD-Induced Autophagy Activation

Upon deprivation of nutrients, autophagy is activated to conserve cellular energy and promote cell survival [1]. Autophagy activation is a complex process and involves a number of regulatory proteins [23] (Supplemental Figure S1). Under stress conditions, the activation of 5′ AMP-activated protein kinase (AMPK) and/or the inhibition of the mammalian target of rapamycin (mTOR) lead to the phosphorylation of ULK1, which initiates autophagy activation. The autophagy activation process includes (1) initiation (ULK1 with FIP200 and ATG13 form the initiation complex); (2) membrane nucleation (Beclin1 and ATG14 are involved in the formation of the PI3K III nucleation complex) and phagophore formation; (3) phagophore expansion (ATG12, ATG5, and ATG16L1 form the ATG12 conjugation system, which promotes the conjugation of LC3. LC3 is cleaved by ATG4 to form LC3-I and then conjugated with phosphatidylethanolamine (PE) to form LC3-II, which interacts with cargo receptors on autophagosome membranes. The level of LC3-II is often used as a marker of autophagy level); (4) fusion with the lysosome; and (5) degradation of LC3-II along with the autophagic cargo [23]. Bafilomycin A1 and leupeptin block autophagosome and lysosome fusion. Because the autophagy process catabolizes the LC3-II protein and results in a paradoxical reduction of LC3-II levels, treatment with Bafilomycin A1 or leupeptin represents an important control to examine the formation of LC3-II.

**Figure 2:**
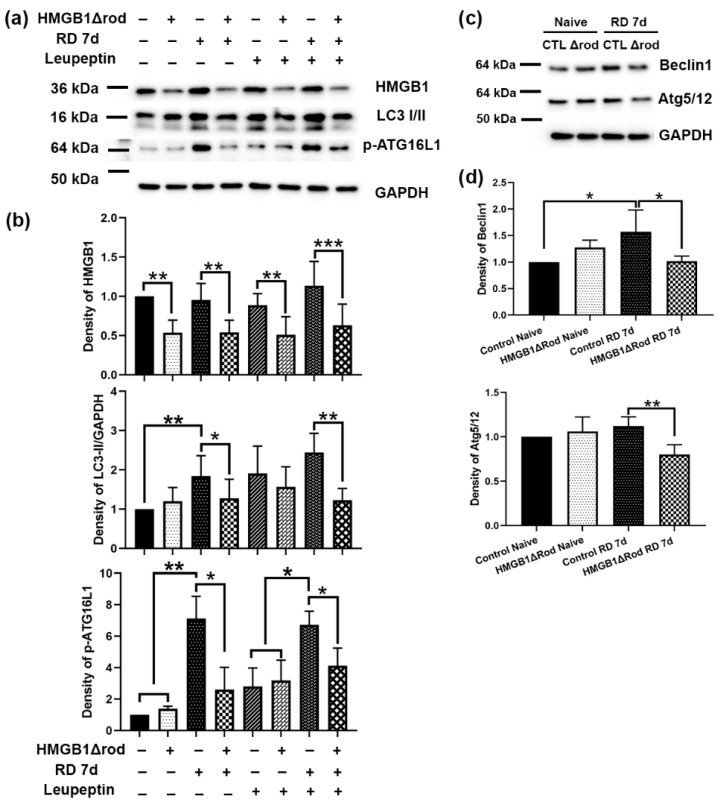
Conditional knock out of HMGB1 reduces autophagy activation following RD. (**a**) Immunoblot analysis of HMGB1, LC3-I/II, and *p*-ATG16L1 in HMGB1Δrod and littermate control mice at 7 dprd, with or without leupeptin treatment to block autophagy flux. (**b**) Graphs show the quantification of protein levels based on densitometry of the Western blots in (**a**). (**c**) Immunoblot analysis of Beclin1 and ATG5/12 complex in HMGB1Δrod and littermate control mice at 7 dprd. (**d**) Graphs show the quantification of protein levels based on densitometry of the Western blots in (**c**). Naïve retinas without manipulation of either eye were used as attached controls. GAPDH serves as a loading control. CTL: control, Δrod: HMGB1Δrod. Data are from three independent experiments and plotted with mean ± s.d. * *p* < 0.05, ** *p* < 0.01, *** *p* < 0.001 (One-way ANOVA).

To assess whether cKO of HMGB1 in rod PR cells affects autophagy activation following RD, we evaluated the levels of regulatory proteins in the autophagy activation pathway in the retinas of HMGB1Δrod mice. As shown in Figure 2a,b, there was significantly less HMGB1 protein in the retinas in HMGB1Δrod mice compared to the controls under both naïve and detached conditions. Similar LC3-II levels were detected in the retinas of control and HMGB1Δrod mice under naïve homeostatic conditions.

Control detached retinas at 7 dprd exhibited markedly enhanced LC3-II levels that were significantly reduced in the detached retinas of HMGB1Δrod mice. In fact, LC3-II levels in the detached retinas of HMGB1Δrod mice at 7 dprd were not significantly different from those of naïve retinas. After blocking autophagy flux with leupeptin, a significant accumulation of the LC3-II protein was apparent in the detached retinas of the control mice. LC3-II accumulation was significantly less in the detached retinas of HMGB1Δrod mice compared to detached controls, suggesting an impairment of autophagy flux in the absence of HMGB1.

The phosphorylation of ATG16L1 by ULK1 at serine 278 is essential for autophagy activation under stress conditions [24], and the level of phospho-ATG16L1 has recently been assessed and found to reliably reflect the rate of autophagy initiation [25]. In keeping with this, phopsho-ATG16L1 was markedly increased after RD in control retinas. Detachment caused a significantly diminished increase in phopsho-ATG16L1 in the HMGB1Δrod retinas, such that its level was not significantly greater than that in naïve retinas. With leupeptin blocking autophagy flux, a slight elevation of phospho-ATG16L1 level was observed in the naïve retinas of both control and HMGB1Δrod mice. In the presence of leupeptin, RD significantly increased the phospho-ATG16L1 level in control retinas, which was significantly blocked in the absence of HMGB1.

The pro-autophagy regulatory proteins Beclin1 and ATG5 can undergo calpain-mediated cleavage to reduce autophagy activation [15,22,26,27]. These truncated products can then shift the cell away from autophagy and toward mitochondria-mediated apoptotic cell death [15,26,27]. We previously demonstrated that the calpain-mediated cleavage of ATG5 peaks at 7 dprd [22]. Thus, we assessed the levels of Beclin1 and the ATG5/12 complex, which are essential for autophagy activation in the retinas of HMGB1Δrod and control mice at 7 dprd (Figure 2c,d). A significant elevation of the Beclin1 level was detected at 7 dprd in control retinas, whereas the lack of HMGB1 in rods abrogated this increase in Beclin1 protein. In addition, cKO of HMGB1 in rods significantly decreased the level of the ATG5/12 complex found in detached retinas.

**Figure 3:**
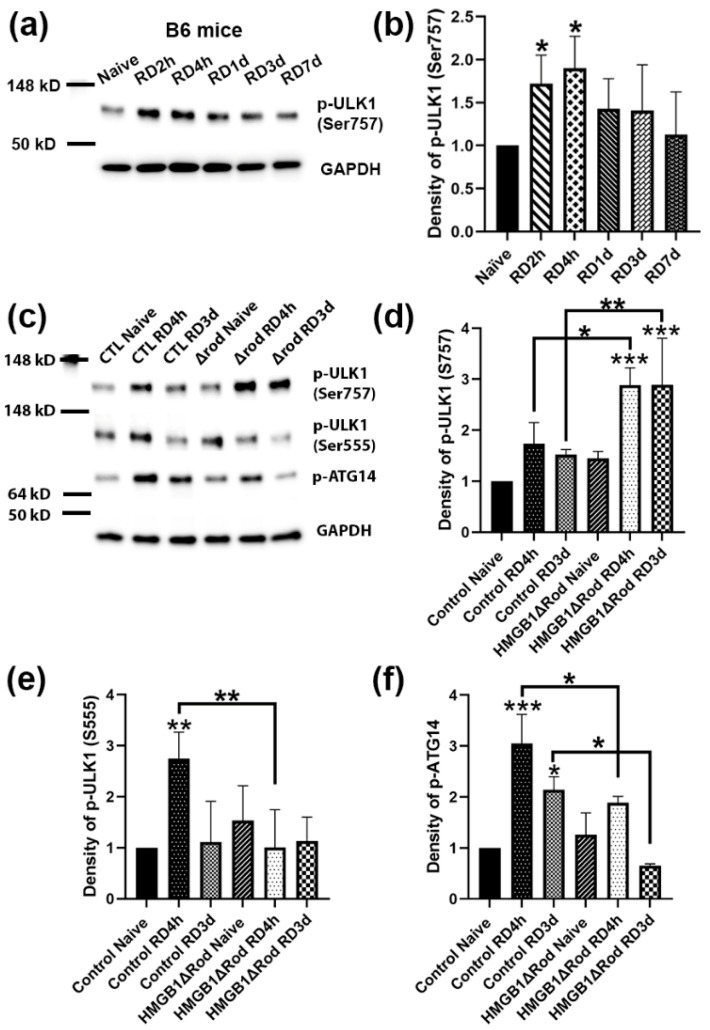
Deletion of HMGB1 shifts the balance away from autophagy activation and toward a pro-inflammation state. (**a**) Immunoblot analysis of p-ULK1 at Serine 757 in B6 mice to evaluate its expression level changes over a time course of 2 h, 4 h, 1 d, 3 d, and 7 d post RD. (**b**) Graph shows the quantification of protein levels based on densitometry of the Western blots in (**a**). (**c**) Immunoblot analysis of p-ULK1 at Serine 757 and 555, and of p-ATG14 at Serine 29 in HMGB1Δrod and littermate control mice at 4 h and 3 d post RD. (**d**–**f**) Graphs show the quantification of protein levels based on densitometry of the Western blots in (**c**). Naïve retinas without manipulation of either eye were used as attached controls. GAPDH serves as a loading control. CTL: control, Δrod: HMGB1Δrod. Data are from three independent experiments and plotted with mean ± s.d. * *p* < 0.05, ** *p* < 0.01, *** *p* < 0.001 (One-way ANOVA). Stars on top of columns represent statistical results compared to the control naïve group.

The phosphorylation of ULK1 serine 555 is critical for autophagy activation [28]. In contrast, the phosphorylation of ULK1 serine 757 by p38α MAPK inhibits its kinase activity and disrupts its interaction with ATG13, reducing autophagy activation [29]. In order to evaluate the phosphorylation of ULK1 following RD, we performed a time-course experiment to detect phospho-ULK1 levels at 2 h, 4 h, 1 d, 3 d, and 7 d post RD. As shown in Figure 3a,b, RD significantly upregulated ULK1 phospho-Ser757 levels at 2 h and 4 h post RD (hprd), which then returned to baseline levels.

We therefore used the two time points, 4 hprd and 3 dprd, to examine how the cKO of HMGB1 in rods affected the ULK1 phosphorylation status after RD. Whereas a lack of HMGB1 protein in rods did not significantly affect ULK1 phospho-Ser757 levels in naïve retinas, detached retinas of HMGB1Δrod mice exhibited the phosphorylation of ULK1 Ser757 at both times, which was significantly greater than in detached retinas of control mice (Figure 3c,d). On the other hand, the deletion of HMGB1 in rods significantly dampened the phosphorylation of ULK1 at serine 555 at 4 hprd (Figure 3c–e). Such a shift from the Ser555 phosphorylation to Ser757 phosphorylation of ULK1 suggests a switch from a pro-autophagy to a pro-inflammatory state.

It is reported that ATG14 is likely to determine the membrane localization of the PI3K complex, and the localization of ATG14 in autophagy-associated membrane requires ULK1 kinase activity [30]. Therefore, we also examined the phosphorylation of ATG14 at serine 29, a ULK1 substrate [31]. ATG14 phospho-Ser29 levels were increased three-fold in control detached retinas at 4 hprd, declining to just over two-fold at 3 dprd (Figure 3c–f). In the detached retinas of HMGB1Δrod mice, phospho-ATG14 levels were significantly diminished both at 4 hprd and 3 dprd compared to detached control retinas at the corresponding time. The results suggest that a lack of HMGB1 in rod PRs inhibited the phosphorylation of ATG14 by ULK1, consistent with the reduced stimulation of autophagy.

Taken together, these data demonstrate that HMGB1 actively supports multiple regulatory steps in autophagy activation following RD, and therefore the lack of HMGB1 in rod PRs reduces autophagy activation and flux.

### 3.3. Rod-Specific Knock out of HMGB1 May Shift the Balance between Autophagy and Inflammation toward a Pro-Inflammatory State

**Figure 4:**
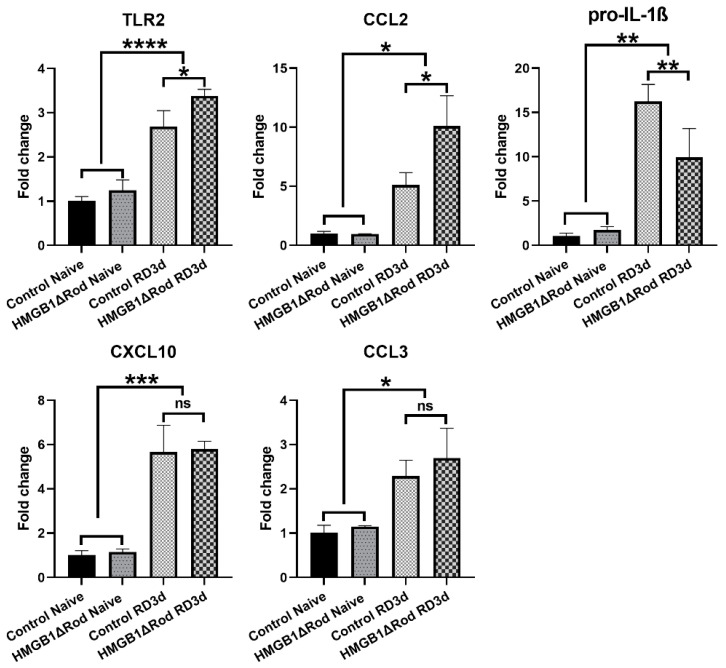
The lack of HMGB1 in rods alters the mRNAs encoding the receptors TLR2, CCL2 chemokine, and pro-IL-1β following RD. The relative levels of TLR2, CCL2, pro-IL-1β, CXCL10, and CCL3 mRNA in retinas of HMGB1Δrod mice and littermate controls were measured by quantitative RT-PCR at 3 dprd. The level of RPL19 mRNA serves as an internal control. Fold changes are relative to littermate control naïve retinas. Data are plotted with mean ± s.d. (*n* = 4). * *p* < 0.05, ** *p* < 0.01, *** *p* < 0.001, **** *p* < 0.0001 (One-way ANOVA). ns: not significant.

Autophagy and inflammation are interrelated. Activation of autophagy negatively regulates inflammation, whereas reduced autophagy correlates with increased inflammation [32]. While the phosphorylation of ULK1 serine 555 stimulates autophagy, the phosphorylation of ULK1 serine 757 promotes the formation of inflammasome [29]. Thus, we hypothesized that rod-specific cKO of HMGB1 could increase the inflammatory response to RD. To assess the inflammatory response between HMGB1Δrod and control groups, we used qRT-PCR to evaluate mRNA levels of inflammation-related genes in the retina at 3 dprd, a time when the number of TUNEL positive cells in the ONL of detached retinas are maximal. As shown in Figure 4, RD significantly increased the mRNA levels of Toll-like receptor 2 (TLR2) and C-C Motif Chemokine Ligand 2 (CCL2) in control retinas, with 2.7-fold and 5.1-fold increases, respectively.

The increases of both TLR2 (3.4-fold) and CCL2 (10.1-fold) mRNAs were significantly enhanced in detached retinas of HMGB1Δrod mice. The mRNA level of pro-interleukin-1β (IL-1β), a major inflammatory cytokine, markedly increased following RD (16.3-fold increase) in control retinas, and this response was significantly dampened in the HMGB1Δrod group (10.0-fold increase). RD also significantly enhanced the mRNA levels of C-X-C motif chemokine ligand 10 (CXCL10) and C-C Motif Chemokine Ligand 3 (CCL3), and a lack of HMGB1 in rods did not significantly impact these responses. Taken together, these data indicate that the cKO of HMGB1 not only dampens the level of autophagy activation, but may also affect the retinal inflammatory response to RD, albeit in an inconsistent manner.

### 3.4. Deletion of HMGB1 in Rods Alters the Expression of Effectors of Both Extrinsic and Intrinsic Apoptosis after RD

**Figure 5:**
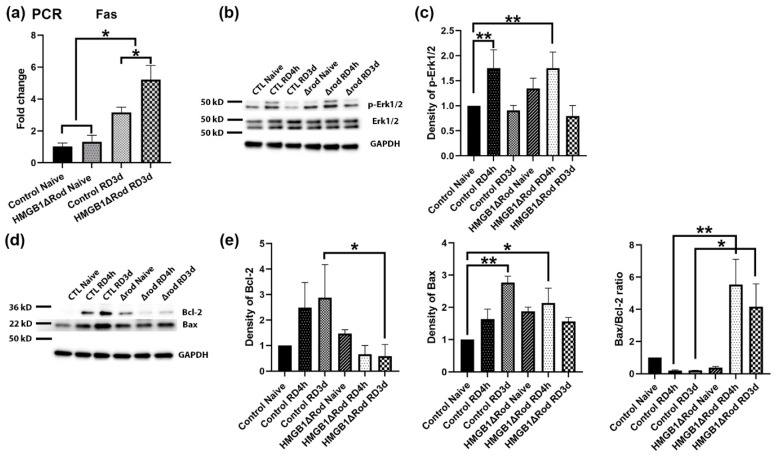
Deletion of HMGB1 in rods causes increased expression of mediators of both extrinsic and intrinsic apoptotic signaling pathways following RD. (**a**) The expression of Fas mRNA in retinas of HMGB1Δrod mice and littermate controls was measured by quantitative RT-PCR at 3 dprd. The level of RPL19 mRNA serves as an internal control. Fold changes are relative to littermate control naïve retinas. (**b**) Immunoblot analysis of p-Erk1/2 and total Erk1/2 protein in retinas of HMGB1Δrod mice and littermate controls at 4 h and 3 d post RD. (**c**) Graph shows the quantification of protein levels based on densitometry of the Western blots in (**b**). (**d**) Immunoblot analysis of Bcl-2 and Bax in retinas of HMGB1Δrod mice and littermate controls at 4 h and 3 d post RD. (**e**) Graphs show the quantification of protein levels based on densitometry of the Western blots in (**d**). Naïve retinas without manipulation of either eye was used as attached controls. GAPDH serves as a loading control. CTL: control, Δrod: HMGB1Δrod. Data are from three independent experiments and plotted with mean ± s.d. * *p* < 0.05, ** *p* < 0.01 (One-way ANOVA).

The death receptor Fas has been shown to be an important extrinsic modulator of PR cell death after RD [33]. We found that RD significantly increased the mRNA expression level of Fas at 3 dprd (3.2-fold) in control retinas and that a lack of HMGB1 in rods further increased Fas mRNA upregulation (5.2-fold) (Figure 5a).

A previous study found that the lack of HMGB1 in mouse embryo fibroblasts increased apoptosis and inhibited autophagy in response to starvation by decreasing the phosphorylation of Erk1/2 and the anti-apoptotic factor Bcl-2, thus sustaining the interaction between Beclin1 and Bcl-2 [14]. We therefore evaluated the effects of HMGB1 cKO on the levels of p-Erk1/2 and proteins in the Bcl2 family, including Bcl-2 and Bax, at 4 h and 3 d post RD. As shown in Figure 5b,c, Erk1/2 phosphorylation increased after RD and the lack of HMGB1 in rods did not significantly affect this response at either time point.

RD also increased total Bcl-2 protein level at both 4 hprd and 3 dprd (Figure 5d,e). However, Bcl-2 proteins were not increased in detached HMGB1Δrod retinas. In fact, Bcl-2 expression at both 4 h and 3 dprd were slightly less than that in naïve retinas. The level of the pro-apoptotic factor Bax was also significantly increased at 3 dprd in the control group. Bax protein levels trended higher in HMGB1Δrod retinas but were not elevated in response to detachment. As a consequence, the ratio of Bax to Bcl-2 (Bax/Bcl-2 ratio) was dramatically and significantly higher in the detached HMGB1Δrod groups compared to controls, indicating that cKO of HMGB1 in rods shifted the balance toward a more pro-apoptotic state after RD.

These results suggest that a lack of HMGB1 in rod PRs may increase cell death after RD not only by hindering autophagy, but also by promoting both extrinsic (Fas-mediated) and intrinsic (mitochondrial permeability transition-mediated) apoptotic pathways.

## 4. Discussion

Retinal detachment disrupts the nutritional support of PR cells from the underlying retinal pigment epithelium, leading to apoptotic PR cell death [18,19,20,21]. Our previous study shows that cKO of HMGB1 in rods causes a more rapid thinning of the ONL [17]. In this study, we further delved into the underlying mechanisms of how HMGB1 affects the survival of PR cells after RD. Our data support our hypothesis that HMGB1 actively promotes autophagy after RD to promote PR cell survival [10]. The conditional KO of HMGB1 in rods impaired autophagy activation, increased the expression of inflammatory genes, and promoted apoptotic pathways, thus leading to more apoptotic PR cell death.

Our previous study demonstrated that RD triggered the translocation of HMGB1 from the nucleus to the cytoplasm [17]. Under starvation conditions, translocated cytoplasmic HMGB1 competitively binds to Beclin1, displacing the inhibitor Bcl-2 from Beclin1 [14]. The interaction between HMGB1 and Beclin1 activates and sustains autophagy, promoting cell survival [14]. In this study, we showed that the cKO of HMGB1 in rods significantly diminished the levels of LC3-II and p-ATG16L1 after RD. Phospho-ATG16L1 recently emerged as a novel reliable marker to measure the rate of autophagy induction, even in the absence of lysosomal inhibitors [25]. These results are consistent with previous studies demonstrating the importance of HMGB1 in regulating autophagy activation, extending them to the rod PR response to RD.

Previous work in our laboratory demonstrated that the level of the ATG5/12 complex, the most important form of ATG5 in autophagy activation, increased at 1 d and 3 d post-RD [22]. However, its level returned to baseline at 7 dprd, likely due to the cleavage of ATG5 by the activation of calpain 1 [22]. Cytoplasmic HMGB1 protects ATG5 and Beclin1 from calpain-mediated cleavage under high-stress conditions [15]. Without HMGB1, these two critical autophagy regulator proteins are cleaved by high levels of calpain, not only compromising autophagosome formation, but also generating proapoptotic proteins. The truncated forms of ATG5 and Beclin1 can cause mitochondria-mediated apoptosis [15,26,27]. The present study showed a significant reduction of both the ATG5/12 complex and Beclin1 at 7 dprd, and increased apoptotic cell death when HMGB1 was conditionally deleted in rods, further supporting the conclusion that HMGB1 is indispensable in stabilizing the ATG5/12 complex and Beclin1 to sustain autophagy and promote cell survival.

There is an increasing literature supporting the relationship between autophagy and inflammation. The activation of inflammasomes in macrophages induces autophagy, which in turn hinders inflammation by targeting ubiquitinated inflammasomes for autophagosome-mediated destruction [34]. In addition, the activation of autophagy in macrophages enhances the lysosomal degradation of pro-IL-1β, hence reducing IL-1β secretion [35]. Blocking autophagy potentiates inflammasome activity and increases inflammatory cytokine production in macrophages challenged with endotoxins [34,36]. In this study, we observed that following RD, a lack of HMGB1 in rods significantly reduced the phosphorylation of ULK1 at serine 555, a modification that promotes autophagy initiation. In the meantime, the lack of HMGB1 in rods enhanced the phosphorylation of ULK1 at serine 757, which inhibits its kinase activity and disrupts its interaction with ATG13, thus reducing autophagy [29]. Furthermore, the phosphorylation of ULK1 at serine 757 by p38 MAPK represents a major mechanism for promoting an inflammatory response [29]. Therefore, the cKO of HMGB1 in rods may shift the balance between autophagy and inflammation toward a pro-inflammatory state. However, only a subset of inflammatory genes were affected, which may be due to expression of these genes in cells other than rod PRs. For example, IL-1β is highly expressed by activated microglia [37], and a lack of HMGB1 in rod PRs may have reduced their response to RD because of HMGB1’s role as an alarmin when released into the extracellular space.

RD causes the activation of both extrinsic and intrinsic signaling pathways, leading to apoptotic PR cell death [33]. This study indicated a greater upregulation of Fas, a key mediator of an extrinsic apoptotic pathway, in the cKO of HMGB1 in rods following RD. The Bcl2 family of proteins play important roles in regulating the intrinsic apoptotic signaling pathway that is triggered by mitochondrial outer membrane permeabilization (MOMP) [38]. An increase in cytoplasmic HMGB1 level is reported to increase the phosphorylation of Erk1/2, which phosphorylates Bcl-2. The phosphorylated Bcl-2 not only dissociates from Beclin1, thus allowing autophagy activation, but also inhibits MOMP [14]. However, our data did not show a significant difference in p-Erk1/2 levels between HMGB1Δrod and control groups at either 4 h or 3 d post RD. Rather, the lack of HMGB1 decreased total Bcl-2 protein expression after RD. This suggests that HMGB1 acts through other pathways to affect the expression of the Bcl-2 protein. Bax is a pro-apoptotic Bcl2 family member that acts as a sentinel for cellular stress [39]. Under stress conditions, Bax relocates to the mitochondrial membrane to initiate MOMP [39,40,41]. The lack of HMGB1 in rods somewhat diminished Bax protein expression after RD, such that, together with the reduction in Bcl-2 expression, the Bax/Bcl-2 ratio was markedly increased in detached HMGB1Δrod retinas. This represents a novel mechanism by which HMGB1 regulates the balance between autophagy and apoptosis during stress conditions.

We used retinal whole-cell lysates to measure autophagy protein changes in retinas from HMGB1∆rod and control groups. We believe that these changes are mainly indicative of differences in rod PRs because cell stress and death following RD occur mainly in the ONL, and rods make up the bulk of PRs in the mouse retina. Furthermore, rod PRs have been found to comprise 66–80% of the total cell population in mouse retinas [42,43], such that whole mouse retina lysates are likely to largely reflect the proteome of these cells. However, a major limitation of our study is that autophagy protein changes in whole retinas may not solely represent changes in rod PRs. Nevertheless, we believe that our transgenic approach is sufficient to demonstrate the biological changes caused by the conditional knock out of HMGB1 in rods. We directly examined the effects of the rod-specific deletion of HMGB1, such that the results can be interpreted as being driven by changes occurring within the rod cells with a fair degree of confidence.

## 5. Conclusions

In conclusion, the present study demonstrates cell-autonomous protective functions of HMGB1 in the rod PR cells. Following RD, HMGB1 actively participates in autophagy activation, suppresses inflammatory responses, and modulates extrinsic and intrinsic apoptotic pathways to promote PR cell survival. These insights into the molecular mechanisms of PR cell survival pathways provide us with new potential opportunities for the development of therapies that prolong PR cell survival in RD and other retinal diseases.

## Data Availability

The data presented in this study are available on request from the corresponding author.

## Supplementary Materials

The following are available online at https://www.mdpi.com/article/10.3390/cells10082010/s1. Figure S1: A simplified schematic diagram depicting the process and main regulatory proteins involved in autophagy activation.
