# Supplementary material for: Conditional Knock out of High-Mobility Group Box 1 (HMGB1) in Rods Reduces Autophagy Activation after Retinal Detachment
Source: Cells. 2021 Aug 6;10(8):2010. doi: 10.3390/cells10082010 (PMC8394251; doi:10.3390/cells10082010)
Supplement: Supplementary file 1 [file cells-1276079-supplementary.pdf › cells-1276079-supplementary.pdf]

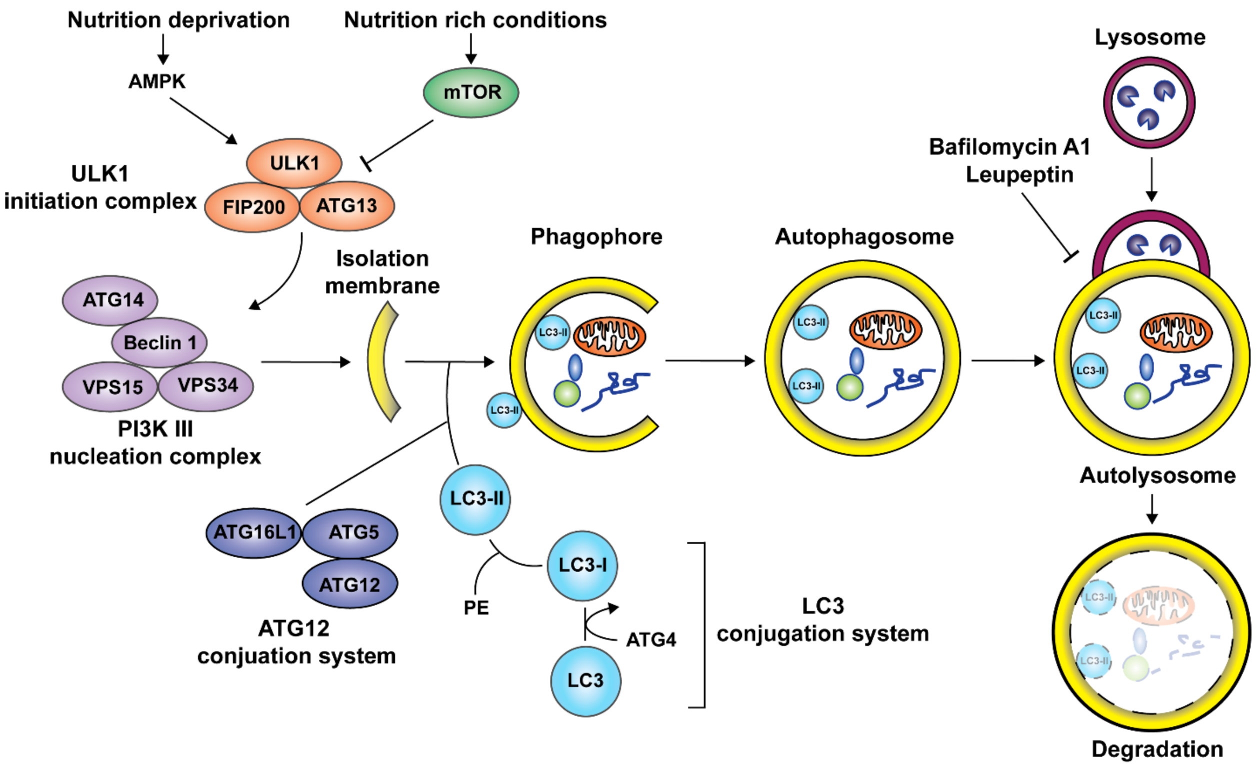

Supplemental Figure S1. A simplified schematic diagram depicting the process and main regulatory proteins involved in autophagy activation. In nutrition-rich state, activation of mechanistic target of rapamycin (mTOR) inhibits autophagy while AMP-activated kinase (AMPK) induced by starvation activates it. The autophagy activation process includes: **1)** Initiation. ULK1 in combination with FIP200 and ATG13 forms the ULK1 initiation complex. **2)** Membrane nucleation and phagophore formation. The PI3K III nucleation complex, comprising of Beclin1, ATG14, VPS15, and VPS34, initiates membrane isolation and promotes phagophore formation. **3)** Phagophore expansion. ATG12 conjugates with ATG5, which is then attached to ATG16L1, forming a ATG12 conjugation system to promotes the conjugation of LC3 system. LC3 is cleaved by the protease ATG4 to form LC3-I, which is conjugated with phosphatidylethanolamine (PE) to form LC3-II. LC3-II is incorporated into phagophore and autophagosome membranes where it interacts with cargo receptors. **4)** Autophagosome formation. Enclosure of phagophore double membranes produces the autophagosome. **5)** Fusion with lysosome. Autophagosome fuses with lysosome to form autolysosome. Bafilomycin A1 and leupeptin are lysosome inhibitors to block the fusion of autophagosome and lysosome, blocking autophagy flux. **6)** Degradation. Engulfed cargo and the inner membrane of autolysosome are degraded, and the resulting nutrients and metabolites are recycled.
